# Aberrant habit formation in the Sapap3-knockout mouse model of obsessive-compulsive disorder

**DOI:** 10.1038/s41598-019-48637-9

**Published:** 2019-08-19

**Authors:** Lotfi C. Hadjas, Christian Lüscher, Linda D. Simmler

**Affiliations:** 1 Department of Basic Neurosciences, University of Geneva, Rue Michel-Servet 1, 1211 Geneva, Switzerland; 2 Service de Neurologie, Department of Clinical Neurosciences, Geneva University Hospital, Rue Gabrielle-Perret-Gentil 4, 1205 Geneva, Switzerland

**Keywords:** Obsessive compulsive disorder, Basal ganglia

## Abstract

Motor behavior can be executed deliberately to achieve specific goals. With repetition, such behavior can become habitual and noncontingent on actions-outcomes. The formation of habits is a natural process that can become pathological, such as in obsessive-compulsive disorder (OCD). The present study used the Sapap3-knockout (KO) mouse model of OCD to assess habit formation based on reward devaluation. We also tested wildtype mice under different training and food-restriction schedules to assess the extent of natural habit formation. We found that Sapap3-KO mice were insensitive to the devaluation of a sucrose reward under conditions in which wildtype littermates were sensitive to devaluation. Moreover, food restriction favored goal-directed action in wildtype mice, whereas mice that were fed *ad libitum* were more likely to form habitual behavior but nevertheless maintained partly goal-directed lever-press behavior. In conclusion, only Sapap3-KO mice developed behavior that was fully insensitive to reward devaluation, suggesting that pathological habits in OCD patients are recapitulated in the present Sapap3-KO mouse model. In wildtype mice, the extent of habit formation was influenced by the state of satiety during training and the reinforcement schedule.

## Introduction

Obsessive-compulsive disorder (OCD) is a mental disorder with a lifetime prevalence of 1–2%^1^. It can cause significant impairment, particularly when considering its onset typically occurs during childhood or adolescence. Inflexible behavior is a typical symptom of OCD^2^, and OCD patients can develop habits more readily than healthy controls^3^. Habitual behavior typically arises after the repetition of action with a particular goal^4^, such as washing hands because they are dirty. In principle, habit formation is a natural and useful process (e.g., habitually washing hands in the bathroom). However, excessively executed habits in the absence of goals, such as frequently washing clean hands, can have negative consequences. Obsessive-compulsive disorder patients may present compulsive behavior that arises from pathological habits. The present study investigated whether aberrant habits are evident in the Sapap3-knockout (KO) mouse model of OCD. Sapap3-KO mice have a constitutive loss of SAPAP3, a postsynaptic density (PSD) protein from the SAP90/PSD-95-associated protein family. The loss of SAPAP3 causes synaptic dysfunction at corticostriatal synapses and OCD-like phenotypes, such as excessive grooming and anxiety^5,6^. Sapap3-KO mice also exhibit impairments in behavioral flexibility^7,8^, indicating that these mice might also present aberrant habit formation.

In rodents, habitual lever pressing can be induced in operant conditioning paradigms by random-interval (RI) training, whereas random-ratio (RR) training favors goal-directed lever pressing^9,10^. Under both training schedules, reward delivery requires lever pressing. Under RI schedules, rewards can be earned in varying time intervals. Under RR schedules, rewards are delivered after varying numbers of lever presses. These two schedules impose different contingencies of response and reward rates and thus facilitate either habitual (RI) or goal-directed (RR) lever pressing. Using these training schedules, the present study sought to determine optimal behavioral conditions for habit formation in wildtype (WT) mice. This was of interest because we found that RI training that resulted in habit formation in Sapap3-KO mice was insufficient for WT littermates to form habits. Testing different variables in WT mice allowed us to appraise habit formation in Sapap3-KO mice within the limits of maximal goal-directed and habitual behavior of WT mice in our laboratory setting.

## Results

### Habit formation in Sapap3-KO mice

**Figure 1:**
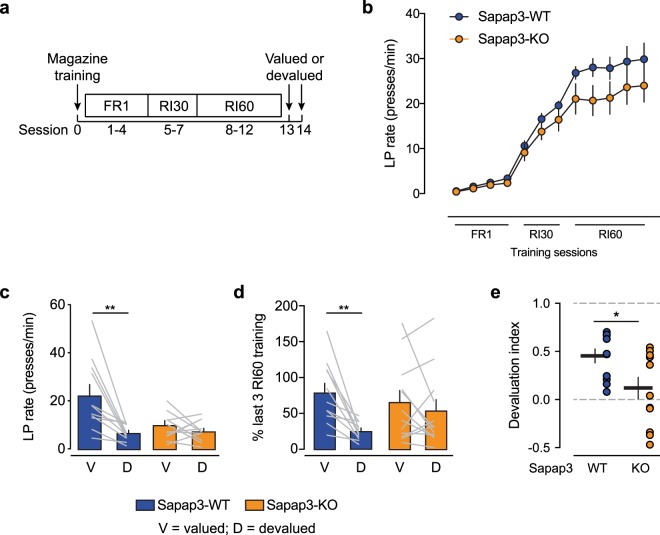
Habit formation in Sapap3-KO mice. (**a**) Behavioral paradigm for habit formation with random interval (RI) training schedules. (**b**) Lever press (LP) rate during training sessions. (**c**) Lever press rate in valued and devalued test sessions. Wildtype littermates but not Sapap3-KO mice exhibited significant reward devaluation. **p < 0.01, valued *vs*. devalued session (paired *t*-test). (**d**) Lever press rates in valued and devalued test sessions normalized to the mean of the last three RI60 training sessions. **p < 0.01 (paired *t*-test). (**e**) Devaluation index, calculated as the following: (LP rate_valued_ − LP rate_devalued_)/(LP rate_valued_ + LP rate_devalued_). A devaluation index of 1 indicates maximal goal-directed behavior. A devaluation index of zero indicates perfectly habitual lever pressing. *p < 0.05 (*t*-test). The data are expressed as mean ± SEM. *n* = 11 WT, *n* = 12 KO.

We trained Sapap3-KO mice and WT littermates to press a lever for a sucrose reward in a relatively short RI training paradigm, with a total of five RI60 training sessions (Fig. 1a). Food consumption and body weight before food restriction did not differ between Sapap3-KO mice and WT littermates (mean ± SD daily food consumption averaged across 4 days: 5.0 ± 1.2 g for Sapap3-KO mice, 4.2 ± 0.6 g for WT mice; mean ± SD body weight: 24.0 ± 3.0 g for Sapap3-KO mice, 24.3 ± 2.9 g for WT mice). Sapap3-KO mice and WT littermates learned the lever press-reward contingency during fixed-ratio 1 (FR1) training and increased their lever press performance with progressive training and according to the imposed training schedule (FR1, RI30, RI60; Fig. 1b). After the completion of training, we used reward devaluation by prefeeding the sucrose reward to test whether the animals had developed habitual lever pressing. For all of the mice, devaluation was controlled for satiety with a separate test session (valued), in which mice were prefed chow but not the sucrose reward. Despite training under an RI schedule, WT mice exhibited reward devaluation, reflected by a significant reduction of the lever press rate during devaluation testing compared with the valued test session (Fig. 1c). In contrast, Sapap3-KO mice did not exhibit reward devaluation, with no significant differences between lever press rates during valued and devalued test sessions. Lever press performance in Sapap3-KO mice was slightly lower during RI training compared with WT littermates. Therefore, we normalized lever press rates for devaluation testing to the last three RI training days (Fig. 1d). The normalized lever press rate did not differ between Sapap3-KO mice and WT littermates, suggesting that the lower lever press rate during valued testing could be explained by generally lower lever press performance in Sapap3-KO mice. The absence of reward devaluation in Sapap3-KO mice suggests that they formed habits under training conditions that were insufficient for WT littermates to form habits. To quantify the degree of goal-directed/habitual behavior, we calculated the devaluation index as the following, based on lever press [LP] rate: (LP rate_valued_ − LP rate_devalued_)/(LP rate_valued_ + LP rate_devalued_). A devaluation index of 1 indicates maximal devaluation (maximal goal-directed behavior). A devaluation index of zero indicates “perfectly habitual”. The devaluation index was significantly different between Sapap3-KO mice and WT littermates (Fig. 1e). The mean devaluation index in Sapap3-KO mice was close to zero, indicating that they developed habitual lever pressing.

### Absence of reward devaluation in Sapap3-KO mice did not result from sensory deficits or a lack of motivation to lever press for sucrose

**Figure 2:**
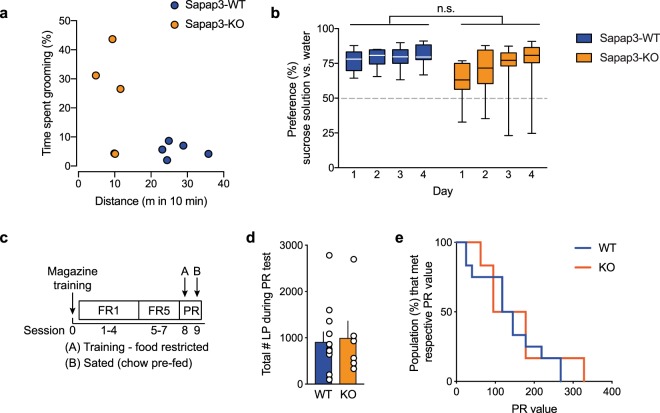
Locomotion, grooming, and sucrose preference in Sapap3-KO mice. (**a**) Distance traveled in the novel open field arena was not correlated with the time spent grooming in Sapap3-KO mice or WT littermates. For the behavioral analysis, the last 10 min of the 30 min session in the open field were scored. *n* = 5 WT, *n* = 5 KO. (**b**) Preference for 10% sucrose solution *vs*. unsupplemented water in the two-bottle choice test. Water and sucrose solution were available in the home cage *ad libitum* for 4 days. *n* = 11 WT, *n* = 9 KO. The two-way repeated-measures ANOVA indicated no significant effect of genotype. Whisker plots show the median, 25th and 75th percentiles, and min/max values. (**c**) Behavioral paradigm for progressive ratio (PR) testing. (**d**) Total number of lever presses in the PR test session after prefeeding chow. The data are expressed as mean ± SEM. *n* = 12 WT, *n* = 6 KO. (**e**) Last PR value achieved in PR testing after prefeeding chow, displayed as the survival curve. *n* = 12 WT, *n* = 6 KO.

The absence of reward devaluation in Sapap3-KO mice indicates that they formed habitual behavior, but it could also be an artefact of behavioral phenotypes that co-exist in these mutant animals. Therefore, we conducted additional behavioral experiments to assess the latter possibility. We first investigated locomotion and grooming in Sapap3-KO mice in a novel open field arena. Sapap3-KO animals were previously reported to groom excessively^5^ and exhibit a reduction of locomotion^8^, but these previous studies were conducted in animals that were ≥4 months old, whereas the mice in the present study were 2–3 months old. We therefore assessed whether locomotion and grooming phenotypes may underlie the lower lever press performance in an operant task. The time spent grooming and distance traveled in the open field were not correlated in Sapap3-KO mice (Fig. 2a). This indicates that they generally exhibited a reduction of the propensity for movement and/or exploratory behavior, which cannot be exclusively explained by their grooming behavior. Despite this phenotype, Sapap3-KO mice learned the operant behavior (Fig. 1), but their lever press performance was slightly lower, probably because of general hypolocomotion and, in some mice, an increase in grooming. However, because of the within-subject comparisons, interference from slow performance does not affect the devaluation test.

Next, we conducted a control experiment to exclude the possibility that the observed habit formation was not an artefact of an inability to sense or establish a preference for sucrose. In a home-cage two-bottle choice test, we assessed the preference for sucrose solution over unsupplemented drinking water. With the exception of one Sapap3-KO mouse, all of the mice exhibited a preference for the bottle with sucrose solution, with no significant difference between Sapap3-KO and WT animals (Fig. 2b). These results indicate that Sapap3-KO mice were able to perceive the palatability of sucrose and preferred the sucrose solution over water, thus strongly suggesting that they were able to process the sensory difference between chow and sucrose pellets in the habit formation task.

Sapap3-KO mice exhibited a reduction of lever press rates during the valued test session compared with WT littermates (Fig. 1c). Thus, we investigated whether differences in the motivation to lever press for a sucrose reward in the satiated (valued) state influenced these results. We trained the mice on FR1 and FR5 schedules and then moved to a progressive-ratio (PR) schedule (Fig. 2c). One day after the PR training session under food restriction conditions, we tested the mice on a PR schedule in a satiated condition (after *ad libitum* chow consumption for 1.5 h). No difference in PR performance was found between Sapap3-KO mice and WT littermates (Fig. 2d,e). The mean breakpoints (±SD) were 156 ± 97 in Sapap3-KO mice and 139 ± 84 in WT littermates. The mean lever press rates were 8.4 ± 7.3 in Sapap3-KO mice and 7.6 ± 6.2 in WT littermates. This experiment indicated that the motivation to lever press for sucrose pellets under satiated conditions, which were the same conditions as for valued testing, did not differ between Sapap3-KO mice and WT littermates.

Altogether, these control experiments suggested that hypolocomotion and an increase in grooming might affect lever press performance in Sapap3-KO mice. However, because these mice exhibited sucrose preference and similar motivation to lever press for sucrose as WT littermates, the insensitivity to reward devaluation was likely attributable to the formation of habits rather than an inherently lower value of the sucrose reward.

### Habit formation in food-restricted WT mice

**Figure 3:**
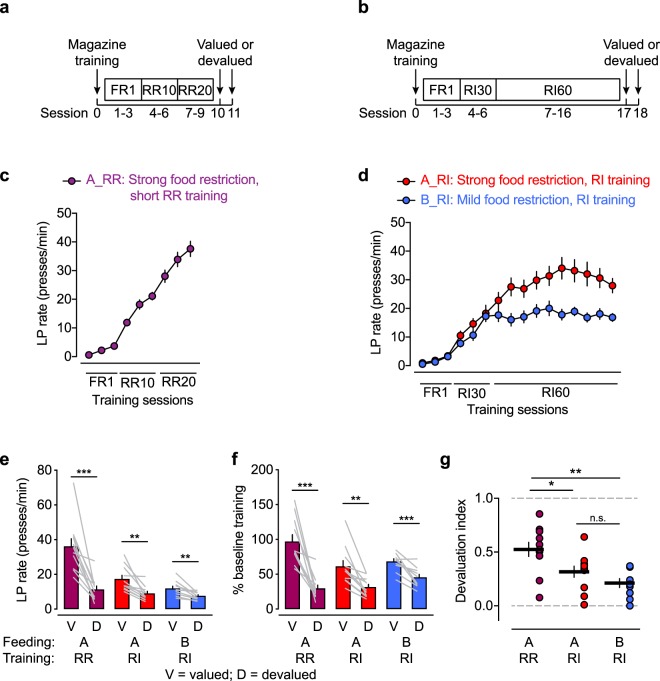
Habit formation in food-restricted WT mice. (**a**) Paradigm to maintain goal-directed behavior under short random-ratio (RR) training schedule. (**b**) Paradigm for the promotion of habitual behavior under a random-interval (RI) training schedule. (**c**) Lever press (LP) rate during short RR training. “A_RR” indicates feeding condition A (strong food restriction) in RR-trained mice. (**d**) Lever press rate during extended RI training. “A_RI” indicates feeding condition A (strong food restriction) in RI-trained mice. “B_RI” indicates feeding condition B (mild food restriction) in RI-trained mice. (**e**) Lever press rate in valued and devalued test sessions. Significant reward devaluation was evident in all training and feeding conditions. **p < 0.01, ***p < 0.001, valued *vs*. devalued session (paired *t*-test). (**f**) Lever press rates from valued and devalued test sessions normalized to the last RR20 training session or to the mean of the last three RI60 training sessions. **p < 0.01, ***p < 0.001, valued *vs*. devalued session (paired Wilcoxon test for A_RR and paired *t*-test for A_RI and B_RI). (**g**) The devaluation index differed significantly between RR- and RI-trained mice but not within RI-trained mice (one-way ANOVA: *F*_2,29_ = 8.431, *p* = 0.0013; Tukey’s multiple comparison test: *p < 0.05, **p < 0.01). The data are expressed as mean ± SEM. *n* = 11 A_RR mice, *n* = 11 A_RI mice, *n* = 11 B_RI mice.

In rats, RI training can induce habitual lever pressing within only a few training sessions, even in the absence of an OCD-like phenotype^9^. However, under the training schedule that we employed for the Sapap3-experiments, WT littermates exhibited reward devaluation that was indicative of goal-directed behavior (Fig. 1). Therefore, we investigated the extremes of goal-directed and habitual behavior in WT mice in our laboratory setting. To maximize goal-directed behavior, we trained WT mice on a short RR training schedule (Fig. 3a). To maximize habitual behavior, we subjected two groups of WT mice to extended RI training (Fig. 3b) that lasted 5 days longer than in the Sapap3-experiment. We hypothesized that strong food restriction hinders the formation of habitual lever pressing because the motivation to lever press in the hungry state is high because of the high caloric value of the reward. We therefore compared strong food restriction (condition A) with mild food restriction (condition B) among WT mice that were trained under an RI schedule. RR mice exhibited a continuous increase in their lever press rate over the training sessions (Fig. 3c). In contrast, RI mice reached a plateau, with mildly food-restricted mice stabilizing at a lower lever press rate than strongly food-restricted mice (Fig. 3d), despite a comparable reinforcement rate (Supplementary Fig. S1a). Across the RI-trained cohorts under food-restriction conditions A and B, the lever press rate during the last three RI60 training sessions was correlated with a reduction of body weight (Supplementary Fig. S1b). Reward devaluation by prefeeding the reward was significant under all three conditions (Fig. 3e), even in RI-trained animals. Similar to the training sessions, lever press rates during the valued test session differed considerably between the training and feeding conditions. Therefore, we normalized lever press rates in the valued/devalued test sessions to lever press rates that were achieved at the end of RR and RI training (Fig. 3f). After the data were normalized, differences between the valued test sessions across groups were less pronounced, thus allowing better discernment of the extent of devaluation under the different conditions. Significant devaluation was also evident after data normalization for all conditions. Nevertheless, the extent of devaluation appeared to be different across conditions. To quantify devaluation, we calculated the devaluation index, which represents the extent of habit formation. Random ratio-trained mice had a significantly higher devaluation index than both RI-trained groups (Fig. 3g). No significant differences in devaluation indices were found between strongly and mildly food-restricted RI-trained animals. Accordingly, the devaluation index was not correlated with the reduction of body weight in RI-trained animals (Supplementary Fig. S1c), indicating that the degree of food restriction affected performance during training but not performance during testing under the valued control condition, in which the mice were tested in the satiated state (i.e., prefed with chow). Nevertheless, the trend in the devaluation index between strongly and mildly food-restricted animals suggests that the extent of food restriction may be a factor that influences the formation of habitual lever pressing.

### Habit formation in *ad libitum*-fed WT mice

**Figure 4:**
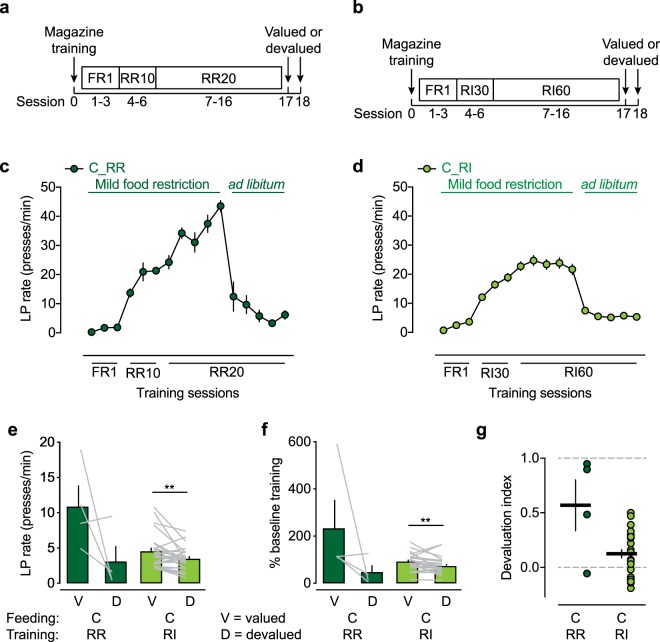
Habit formation in WT mice fed *ad libitum*. (**a**) Paradigm to maintain goal-directed behavior under an extended random-ratio (RR) training schedule. (**b**) Paradigm for the promotion of habitual behavior under an extended random-interval (RI) training schedule. (**c**) Lever press (LP) rate during extended RR training. “C_RR” indicates feeding condition C (mild food restriction followed by *ad libitum* feeding) in RR-trained mice. (**d**) Lever press rate during extended RI training. “C_RI” indicates feeding condition C (mild food restriction followed by *ad libitum* feeding) in RI-trained mice. (**e**) Lever press rate in valued and devalued test sessions. Significant reward devaluation was evident in RI-trained mice. **p < 0.01, valued *vs*. devalued session (paired Wilcoxon test). (**f**) Lever press rates from valued and devalued test sessions normalized to the last RR20 training session or to the mean of the last three RI60 training sessions. **p < 0.01, valued *vs*. devalued session (paired Wilcoxon test). (**g**) The devaluation index did not differ significantly between RR- and RI-trained mice (*p* > 0.05, *t*-test with Welch’s correction for unequal standard deviation). The data are expressed as mean ± SEM. *n* = 4 C_RR mice, *n* = 26 C_RI mice.

Wildtype mice that were trained under mild food restriction conditions exhibited reward devaluation, suggesting that their lever press behavior was to some extent still goal-directed, despite RI training. Therefore, we investigated next whether even mild food restriction during RI training impedes habit formation. We trained a cohort of WT mice on the extended RI schedule (Fig. 4b) but stopped food restriction after day 5 of RI60 training. Because caloric need was no longer a motivating factor for training, the lever press rate dropped significantly when the mice were placed on *ad libitum* feeding, and they maintained lever pressing behavior on an RI60 schedule at a lower lever press rate (Fig. 4d). To control for goal-directed behavior, we trained a group of mice on an extended RR schedule (Fig. 4a), in which the number of training days matched the extended RI schedule. Similar to RI-trained mice, RR-trained mice exhibited a significant decrease in lever press performance after they were placed on *ad libitum* feeding but maintained lever pressing behavior until the end of training (Fig. 4c). Because the lever press rate was low in *ad libitum*-fed mice, we extended the devaluation test sessions from 5 to 10 min and excluded all mice that did not lever press at least 20 times on the active lever during the valued test session. Of the 11 *ad libitum*-fed mice that we trained on the RR schedule, only four remained after applying the exclusion criterion. In the RI-trained cohort, seven of 33 mice were excluded because of their valued testing performance. Three of the four remaining RR-trained mice exhibited clear reward devaluation (Fig. 4e,f). The RI-trained mice still exhibited reward devaluation at the group level, assessed by paired *t*-tests (Fig. 4e,f), but they exhibited a high degree of habitual behavior, reflected by a devaluation index that approached zero (Fig. 4g), indicating that these mice exhibited mostly habitual lever pressing. In contrast, RR-trained mice (Fig. 4g) had a devaluation index that was comparable to mice that were trained on the RR schedule under strong food restriction conditions (Fig. 3g). These data indicated that extended RR training in *ad libitum*-fed mice promoted goal-directed behavior, whereas extended RI training in *ad libitum*-fed mice promoted habit formation. Furthermore, *t*-test comparison indicated that the devaluation index in RI-trained, *ad libitum*-fed mice was significantly less than in strongly food-restricted mice (*p* < 0.01, feeding condition C in RI-trained mice *vs*. feeding condition A in RI-trained mice). Among all of the variables that we tested in WT mice, extended RI training, combined with *ad libitum* feeding, yielded the most extreme habitual behavior, with a devaluation index that approached zero.

## Discussion

The present study tested the propensity of Sapap3-KO mice (i.e., a mouse model of OCD) to develop habitual lever press behavior in a classic habit-formation operant task, based on reward devaluation. Sapap3-KO mice exhibited insensitivity to reward devaluation, which is indicative of habitual behavior, under conditions that did not induce habit formation in WT littermates. Furthermore, extended RI training and a lower degree of food restriction promoted habit formation in WT mice, but some degree of residual goal-directed behavior remained in all WT animals.

Aberrant habit formation has been suggested to be a clinical symptom of OCD^3^. Compared with control subjects, OCD patients are more prone to respond to a stimulus despite devaluation, suggesting that they readily form habitual behavior in a laboratory setting^11^. The present study reproduced this clinical symptom in an animal model of OCD. Our findings support the validity of Sapap3-KO mice as a translational animal model of OCD and endorse the future use of Sapap3-KO mice to test treatments that seek to alleviate aberrant habits in OCD. The present study is the first to report that Sapap3-KO animals form habits more readily than WT littermates. However, two recent studies reported that cognitive flexibility, tested by reversal learning tasks, was impaired in Sapap3-KO animals^7,8^. Inflexibility to adjust behavior, as measured in reversal learning tasks, might be related to aberrant habit formation in this OCD model^2^.

The neuronal mechanisms that underlie aberrant habit formation in Sapap3-KO mice have not yet been investigated. Corticostriatal dysfunction has been described as a consequence of the loss of the PSD protein SAPAP3^5,6,12–15^. The dorsal striatum, which receives strong inputs from different cortical areas, has been implicated in the progression from goal-directed behavior to habitual responses^16^. Therefore, the knockout of *Sapap3* might disturb synaptic signaling that is involved in the cortical control over striatal outputs that determine goal-directed or habitual actions. By assessing optogenetically evoked corticostriatal synaptic responses, Corbit *et al*.^14^ proposed a model in which the medial striatum in Sapap3-KO animals receives less inputs from the orbitofrontal cortex compared with WT littermates. Weak orbitofrontal cortex inputs might underlie the insensitivity to reward devaluation in Sapap3-KO animals. In WT animals that formed habits, signaling from the orbitofrontal cortex projection to the striatum was synaptically depressed^10,17^. However, the inactivation of other cortical areas, such as the prelimbic cortex^18,19^ and premotor cortex^20^, also results in insensitivity to reward devaluation, suggesting that the function of these cortical areas is also important for normal goal-directed actions. The constitutive knockout of *Sapap3* likely affects corticostriatal projections in general. Therefore, dysfunctional synaptic control from the prelimbic and motor cortices may contribute to the habit formation phenotype in Sapap3-KO animals. Further research is needed to elucidate specific corticostriatal dysfunction and its relationship to aberrant habit formation in this animal model of OCD.

In the present study, we found that the induction of habitual lever pressing in WT mice was more susceptible to failure compared with Sapap3-KO mice. Wildtype mice trained under an extended RI schedule, which is known to favor habit formation^9^, still exhibited significant devaluation, although to a lesser extent than WT animals that were trained under RR schedules. We hypothesized that under conditions of food restriction, caloric need during training prevents or hampers the transition from goal-directed behavior to habitual lever pressing behavior. Indeed, the devaluation index in RI-trained mice that were fed *ad libitum* was significantly lower than in mice that were trained under strong food restriction conditions. This suggests that feeding conditions or any state that interferes with reward perception in laboratory studies with mice can variably influence habit formation. When mice are trained under conditions in which the reward is needed, such as when the caloric value of the reward is essential, the goal-directed component of behavior is more prevalent than when mice are trained under conditions of *ad libitum* feeding, in which they consume the reward for its palatability rather than its caloric value.

A potential limitation of the present study could be a change in valued control sessions, which then would impact on devaluation indices. The different training and feeding conditions that were tested in WT animals resulted in differences in performance during training, which was then also reflected by different valued lever press rates. However, the devalued lever press rates did not differ as much across conditions as did the valued lever press rates, suggestive of a floor effect. Nevertheless, even in the more habitual groups with low lever press rates during valued testing, all of the mice pressed the active lever during the devaluation session. A true floor effect would suggest that devalued mice would stop lever pressing completely. Furthermore, previous studies that assessed habit formation in mice^17,21^ and rats^9^ also found that the main difference between non-habitual and habitual animals is manifested in valued test sessions and not in devalued test sessions.

In conclusion, we found that Sapap3-KO mice readily formed habits, whereas the extent of reward devaluation in WT mice was influenced by the duration of training, reinforcement rate, and food restriction. The habits developed by the Sapap3-KO mice appeared aberrant, reflecting inflexibility in their behavior that mimics symptoms in OCD patients.

## Methods

### Animals

Sapap3-mutant mice were obtained from Dr. Gouping Feng (Massachusetts Institute of Technology). The mice were backcrossed on a C57BL/6J background for >20 generations. Sapap3^−/−^ (KO) mice and Sapap3^+/+^ mice (WT littermate controls) were generated from Sapap3^+/−^ breeding. Male and female Sapap3-KO and Sapap3-WT mice were used. For the experiments with WT mice only, male C57BL/6J mice were purchased from Charles River. The experiments were conducted with 7- to 15-week-old mice. The animals were housed under a normal 12 h/12 h light/dark cycle (lights on at 7:00 AM). Water was provided *ad libitum*. Food was provided either *ad libitum* or restricted during operant training. All procedures were approved by the Institutional Animal Care and Use Committee of the University of Geneva and by the animal welfare committee of the Canton of Geneva, in accordance with Swiss law.

### Habit formation: Sapap3

Operant training began with Sapap3-KO mice and WT littermates at 7–12 weeks of age. The animals were food restricted to a maximum of 85% body weight with 2.0 g chow per day. All of the operant sessions began by turning on the house light in the operant boxes and the ventilation fan in the sound-attenuating chambers (Med Associates). The mice underwent one session per day. In session 0, the mice underwent magazine training, during which no levers were available in the chamber, and a sucrose reward (50% sucrose pellets, 20 mg, TestDiet, 5TUL, catalog no. 1811142) was dispensed on an RI60 schedule (one reward dispensed on an average of every 60 s) for 10 min. The mice were left in the operant boxes for an additional 20 min for habituation. In sessions 1–4, the mice were trained on an FR1 schedule to press the active lever to receive a reward. A second, inactive lever was present in the box. In sessions 5–7, the mice were trained on an RI30 schedule (every 3 s, lever pressing was rewarded at a 10% probability). In sessions 8–12, the schedule was RI60 (every 6 s, lever pressing was rewarded at a 10% probability). The training sessions ended when 30 rewards were earned or when 60 min elapsed within a session. On valued and devalued testing days (days 13 and 14, counterbalanced), the mice were fed *ad libitum* for 1.5 h with either chow (valued) or sucrose pellets (devalued). The mice were transferred to the operant boxes immediately after feeding. During the 5 min valued/devalued test sessions, both levers were present in the box, but lever pressing did not result in a reward. The active and inactive levers and the order of valued and devalued test sessions were counterbalanced within each group of mice.

### Habit formation: WT cohorts

The habit formation task was conducted with WT animals using the same procedure as with Sapap3-KO mice and their WT littermates but with the following modifications. Operant training began at 10 weeks of age. Three different food restriction regimens and three different training schedules were used to assess the effects of these variables on habit formation. Mice that were subjected to food restriction condition A (“strong food restriction”) were fed 2.0 g chow per day. Mice that were subjected to food restriction condition B (“mild food restriction”) were fed 2.25 g chow per day until session 5 and then 2.75 g chow per day. Mice that were subjected to food restriction condition C were mildly food restricted (see condition B) until the completion of training session 11 and then maintained on chow *ad libitum* in sessions 12–18. Operant training deviated from the procedure that is described above (*Habit formation: Sapap3* section) as WT cohorts were trained on FR1 for only three sessions. WT mice were then trained on a short RR schedule (RR10 in sessions 4–6, RR20 in sessions 7–9), extended RR schedule (RR10 in sessions 4–6, RR20 in sessions 7–16), or extended RI schedule (RI30 in sessions 4–6, RI60 in sessions 7–15). Under the RR10 schedule, every lever press was rewarded at a 10% probability. Under the RR20 schedule, every lever press was rewarded at a 5% probability. Devaluation testing was performed as described for Sapap3 on the 2 days following the completion of RR or RI training. The RR and RI training sessions were terminated after 30 rewards were earned or when 60 min elapsed, with the exception of food restriction condition C, in which the sessions lasted a maximum of 90 min. Devaluated and valued control sessions had a 5 min duration, with the exception of food restriction condition C, in which the sessions lasted 10 min. Mice that performed <2 lever presses per minute in the valued test session were excluded (in food restriction condition C: 7 of 11 mice in the RR group and 7 of 33 mice in the RI group).

### Progressive ratio

Sapap3-KO mice and WT littermates were food restricted to a maximum of 85% body weight with 2.0 g chow per day. All of the operant sessions began by turning on the house light and ventilation fan in the sound-attenuating boxes. In session 0 of the operant task, the mice underwent magazine training (see details under *Habit formation* above). In sessions 1–4, the mice were trained to press the active lever for a reward on an FR1 schedule. Pressing the inactive lever was not rewarded. Rewards were available on an FR5 schedule in sessions 5–7. For all days of training, the sessions ended when the mice earned 30 rewards or when 60 min elapsed. In session 8, the mice underwent PR training, in which lever pressing was rewarded on a PR schedule, calculated as 5 × e^(*R*×0.2)^ − 5, where *R* is the number of pellets already earned^22^. On the day of PR testing (session 9), the mice were provided with chow *at libitum* for 1.5 h in their home cages before the operant session, and PR responding was then tested under satiated conditions. For PR testing, the session ended when a maximum time of 120 min elapsed or when no lever press occurred in 20 min. The active and inactive levers were counterbalanced within each group of mice.

### Sucrose solution/water choice test

The mice were singly housed and allowed 3 days for habituation to isolation. A water bottle that was supplemented with 10% (w/v) sucrose was then provided, in addition to a bottle with unsupplemented water. The mice had access to both bottles for 4 days. Consumption was recorded by weight after the first night and then every 24 h thereafter.

### Locomotor activity and grooming

Five Sapap3-KO mice and five Sapap3-WT littermates were tested for locomotion and grooming in a novel open field 2 days after they completed the habit formation task. On the day of open field testing, the mice were transferred to the behavior room and left for habituation for >30 min. The mice were then separately placed in a 35 cm × 35 cm arena with moderate illumination (80–100 lux at the bottom of the arena) for 30 min. A video camera was located above the arena to record the sessions. Using AnyMaze software, locomotor activity was analyzed as the distance travelled during minutes 20–30 of the session. Grooming was manually scored during minutes 20–30 of the session by an experimenter who was blind to genotype. Only grooming bouts with a duration of >2 s were included.

### Statistical analysis

The data were tested for a normal distribution using the Shapiro-Wilk test. Parametric tests were used for normally distributed data. Nonparametric tests were used for data that did not pass the normality test. Significant main effects in the analysis of variance (ANOVA) were followed by *post hoc* tests. Comparisons between two variables and *post hoc* tests were two-tailed with a significance level of 5%. Details of the statistical tests and significance levels are provided in the figure legends.

## Supplementary information

Supplementary Dataset 1

## Data Availability

The data are available from the corresponding author upon request.
