# Supplementary material for: Aberrant habit formation in the Sapap3-knockout mouse model of obsessive-compulsive disorder
Source: Sci Rep. 2019 Aug 19;9:12061. doi: 10.1038/s41598-019-48637-9 (PMC6700199; doi:10.1038/s41598-019-48637-9)
Supplement: Supplementary file 1 — Supplementary Dataset 1 [file 41598_2019_48637_MOESM1_ESM.pdf]

Aberrant habit formation in the Sapap3-knockout mouse model of obsessive-compulsive disorder

Lotfi C. Hadjas, Christian Lüscher, and Linda D. Simmler

Supplementary Information

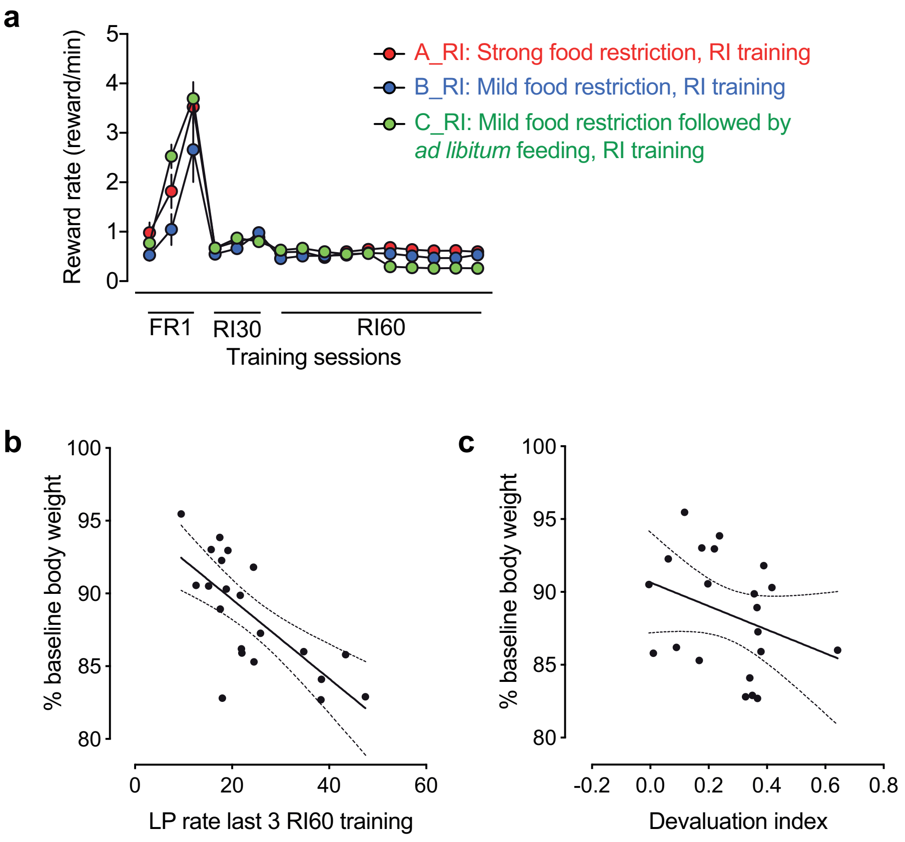

Supplementary Figure S1. Additional analyses from the habit formation task in WT mice. (a) Reward rates during FR1 and random interval (RI) training in feeding conditions A (strong food restriction), B (mild food restriction), and C (mild food restriction followed by *ad libitum* feeding). **(b)** Scatter plot of the reduction of body weight (average from last three training days, expressed as a percentage of baseline before food restriction) *vs.* lever press

(LP) performance (LP/min) during the last three RI training sessions. Data from cohorts A and B that were trained on an RI schedule were taken into account. Body weight and LP rates during training were significantly correlated (Pearson's r = -0.72, $p < 0.001$). $n = 21$. **(c)** Scatter plot of the reduction of body weight (same as in B) *vs*. devaluation index. Data from cohorts A and B that were trained on an RI schedule were taken into account. Body weight and devaluation indices were not correlated (Pearson's r = -0.33, $p = 0.15$). $n = 21$.
